# Exploring the value of community engagement activities within a participatory action research study to improve care for people affected by skin neglected tropical diseases in Liberia

**DOI:** 10.1186/s40900-025-00695-2

**Published:** 2025-03-24

**Authors:** Maisy Lopez Piggott, Zeela Zaizay, Laura Dean, Georgina Zawolo, Coleen Parker, Rosalind McCollum

**Affiliations:** 1 Department for International Public Health, Liverpool School of Tropical Medicine, Liverpool, L3 5QA UK; 2 Actions Transforming Lives, Congo Town Backroad, Monrovia 1000, Liberia; 3 Pacific Institute for Research and Evaluation, University of Liberia, 1000 Monrovia, Liberia; 4 Research Department, Ministry of Health-Liberia, Monrovia, Liberia

**Keywords:** Community engagement, Skin neglected tropical disease, Participatory research, Value creation, Dual role researchers

## Abstract

**Background:**

Skin Neglected Tropical Diseases (NTD) can impact physical and mental well-being for persons affected due to discrimination and stigmatisation, often leading to feelings of disempowerment. Community engagement is important for NTD work to foster advocacy and empowerment; however, there is limited literature surrounding best practices for community engagement within research focused on skin NTDs. REDRESS is a participatory action research study, aimed at reducing the burden of skin NTDs through a person-centred approach that emphasises community engagement. This study explores the value of community engagement within REDRESS for individuals and the impact on the health system’s ability to care for person’s affected by skin NTDs.

**Methods:**

Through a naturalistic paradigm, eleven purposively selected in-depth interviews and 21 in-depth interviews with reflective diary participants were conducted in Liberia. Participants included peer-researchers, co-researchers, and dual role participants (Ministry of Health implementers and REDRESS researchers). Taking an inductive epistemological position, data was thematically analysed around a value creation framework that considers different cycles of value creation for communities such as potential, immediate and transformative value.

**Results:**

This study revealed that REDRESS community engagement aligned with core UNICEF community engagement standards and identified seven themes relating to value creation cycles, participant position and enabling environments. Community engagement led to capacity building and collaboration, provided communities tools to make changes and share knowledge, and had transformative effects in trust and health seeking behaviours. The primary research revealed community engagement activities not seen elsewhere such as the involvement of persons affected as peer-researchers and communities taking part in project monitoring through keeping reflective diaries. Researchers’ local involvement facilitated processes that would not otherwise occur, such as speaking local dialects and policy discussions. Importantly, power dynamics are carefully considered in the process. Few challenges have been discussed directly related to REDRESS, but individual challenges related to dual role time management, external factors, and differences in agendas.

**Conclusion:**

Community engagement activities led to meaningful empowerment, ownership, sustainability, and partnership formation leading to broader health outcomes. Five areas of opportunity were identified, and recommendations to strengthen community engagement include capacity building, clearer communication and addressing power imbalances.

**Supplementary Information:**

The online version contains supplementary material available at 10.1186/s40900-025-00695-2.

## Abstract (plain language summary)

Skin neglected tropical diseases (NTDs) significantly impact physical and mental health, contributing to stigmatisation and social exclusion. REDRESS, a multidisciplinary participatory action research study focuses on strengthening care for people affected by skin NTDs in Liberia, through collaboration with persons affected and communities. To date, research on the role of community engagement in participatory action research studies seeking to improve care for persons affected by skin NTDs remains limited. In response, we consider the value of involvement within REDRESS for different communities, including people affected by skin NTDs. Interviews were conducted with eleven participants within REDRESS, including those with a dual role (ministry of health implementers and REDRESS researchers) alongside 21 in-depth interviews with participants who kept diaries reflecting their involvement within the study (affected individuals, formal and informal health providers). A framework looking at value creation from engagements was used to underpin this. We find that direct engagement with beneficiaries from REDRESS added positive value to the work. Communities learned new skills and collaborated with researchers, improving their trust in the health system and health seeking behaviours. The involvement of person’s affected by skin NTDs and local health workers as researchers was positive. However, challenges such as time management, unmet expectations, and external factors such as poor road conditions were noted. Working alongside communities empowered participants, regained control, and achieved broader health outcomes. Managing power dynamics in participatory studies is crucial, and to further strengthen engagement a focus on skill development, clear communication, and reducing power imbalances is essential.

**Supplementary Information:**

The online version contains supplementary material available at 10.1186/s40900-025-00695-2.

## Background

Skin neglected tropical diseases (NTDs) are a type of NTD that presents with visible characteristics on a person’s skin. Some NTDs can be classified within a wider group of skin conditions due lesions appearing on the skin or manifesting themselves through skin changes long before any evident impact on internal organs [1, 2]. Skin NTDs are estimated to affect more than 900 million people worldwide [3]. This disease burden is mostly located in tropical and subtropical areas, affecting vulnerable and disadvantaged populations who often experience significant socioeconomic inequalities [4, 5]. Skin NTDs can negatively impact the psychosocial well-being and quality of life of persons affected, resulting from internalised and experienced stigma, as well as the physical impact of the conditions such as long-term disfigurement and disability [6–9].

There are at least 9 skin NTDs identified by the World Health Organisation (WHO) [10], listed below:

1. Yaws
2. Leprosy (Hansen disease)
3. Onchocerciasis
4. Lymphatic filariasis (lymphoedema and hydrocele)
5. Buruli ulcers
6. Cutaneous leishmaniasis
7. Mycetoma, chromoblastomycosis, and other deep mycoses (including sporotrichosis)
8. Post kala-azar dermal leishmaniasis
9. Scabies and other ectoparasitoses (including tungiasis)

The World Health Organisation (WHO) 2030 NTD Road Map [5] emphasises that community-based research is “*essential for a building solid foundatio*n” for effective NTD interventions and group empowerment. However, despite the increasing emphasis on community-based research, there is limited evidence on the value of community engagement approaches among persons affected by skin NTDs, and others involved in skin NTD care.

Although no standard definition of “*community*” exists, it is understood to be a shared common identity, such as geography, faith, culture or health [11]. Community engagement in research is “*the meaningful, respectful, and fit-for-purpose involvement of community members in one or more aspects of research projects*” [12]. Community engagement aims to involve communities/groups of people in addressing issues that affect them, thus promoting empowerment, stronger networks and building capacity [13]. Community engagement exists along a continuum, ranging from tokenism to shared leadership.

Traditionally, researchers collect data from participants who have minimal input [14]. Recently, guided by the WHO NTD roadmap, there has been a shift towards more active community involvement throughout research cycles of NTD research programs [5]. However, high levels of stigma and discrimination associated with skin NTDs can hinder community engagement efforts, creating barriers for persons affected to seek healthcare services and participate within the community [15].

Power dynamics, or the ability to influence or control outcomes, plays an important role in research processes and findings [16]. Dimensions of power are seen through spaces (closed, invited or claimed), forms (visible, hidden or invisible) and levels (global, national or local) [17]. Thus, considering preexisting power hierarchies within society is central to effective community engagement, ensuring that affected communities are meaningfully involved as decision-makers throughout the research process [18].

### Liberian context and role of community engagement within REDRESS

Liberia, a low-income country on the West Coast of Africa, is home to more than 5.18 million people, half of whom lived below the national poverty line in 2016 [19]. Liberia’s complex sociopolitical history, including 14 years of civil conflict and a devastating Ebola epidemic, severely weakened its fragile health system [20]. Liberia is endemic for a range of skin NTDs, including leprosy, Buruli ulcer, yaws, lymphatic filariasis and onchocerciasis. The Liberia Ministry of Health (MOH) established the NTD program in 2011, and Liberia was among the first countries to introduce integrated case management of NTDs in 2016 through the *“Strategic Plan for Integrated Case Management of Neglected Tropical Diseases 2016–2020”* [21].

REDRESS [22], an implementation research consortium, involves the Liberia MOH, research institutes in the United Kingdom and Liberia and nongovernmental organisations (NGOs) experienced in NTD care.^1^ This multidisciplinary project aims to reduce the burden of skin NTDs via a “*person-centred approach to evaluate, develop and adapt health systems interventions for the management of severe stigmatising skin diseases (SSSDs) in sub-Saharan Africa*” [23], by using Liberia as an intervention site for transferable knowledge [24]. The skin NTDs endemic to Liberia and studied within REDRESS include leprosy, Buruli ulcer, yaws, lymphatic filariasis and onchocerciasis [25].

The REDRESS project encompasses five research cycle phases described below [23], of which the first two were completed at the time of this study:

1. Formative research: assessment of current integrated approaches for the early identification, referral, and treatment of SSSDs aligning with key focus areas.
2. Planning: collaboration with health system actors and individuals affected to develop new interventions and refine existing interventions.
3. Action: delivery of codeveloped interventions within existing health systems and community frameworks.
4. Observation & reflection: multidisciplinary assessment of the integrated interventions, providing recommendations for quality enhancement and the expansion of these interventions.
5. Knowledge translation & policy change: working alongside the MoH throughout the duration of REDRESS to expand and integrate effective interventions, and provide recommendations on these across the West African region.

The REDRESS [18] community engagement strategy has identified a range of communities, including patients, health workers, decision makers, the general population and other National Institute for Health and Care Research (NIHR) funded groups, as central to engagement activities. These communities have been engaged through roles such as peer-researchers, co-researchers and through community advisory boards (Table 1). Participatory methods (such as Photovoice, Stepping Stones and the use of Vignettes) have been used to ensure the inclusion of typically unheard voices [15, 26].

**Table 1:** Description of roles within REDRESS related to community engagement

Role	Description of role
Peer-researchers	Peer-researchers in REDRESS, included people affected by skin NTDs, community health care workers (community health: assistants, volunteers, or service supervisors), faith and traditional healers, and health systems NTD actors During the intervention phase, 48 peer researchers kept reflective diaries to document changes in communities. At other study phases (formative and evaluation) peer-researchers were involved with photovoice research
Co- researchers	A total of 8 co-researchers across the three intervention counties, including community health workers (community health assistants and community health service supervisor assuming a dual role) and a person affected by skin NTD. As part of the core research team, they played a role in research data collection, analysis and dissemination following REDRESS training. Travel expenses during the research period were reimbursed
Community advisory board (CAB)	The community advisory board was composed of representatives within community structures, these included persons affected by skin NTDs, people with disabilities, community health assistants, traditional healers, faith healers, women’s groups, youth groups, agriculture groups and people with mental health conditions, as well as health workers and local health decision makers. In meetings, board members contributed insights and guidance to REDRESS ensuring the project objectives aligned with local and national priorities
Dual role	This refers to Ministry of Health (MOH) actors involved with implementing NTD programme activities at county and national levels, as well as playing role within REDRESS. For some at national level, this was alongside their current MOH jobs. These roles included supporting research design and leading research development planning and implementation Additionally, some MOH formed part of the MOH technical advisory board and took part in various technical working groups, established to progress development of the REDRESS intervention
Person affected	Persons affected by a skin NTD such as Leprosy, Buruli Ulcer, Yaws, Lymphatic Filariasis and Onchocerciasis
Traditional and faith healers	Informal health providers caring for people affected within communities through country medicine (herbs and leaves) and faith
Reflective diary	Participatory method which involves recording a person’s experiences and why these are important, as well as feelings and challenges

Comprehensive community engagement is important for fostering advocacy, empowerment, and positive self-perception, which are deemed beneficial outcomes in facilitating marginalised persons or populations to rejoin society. Consequently, UNICEF [13], the WHO [27] and the Centers for Disease Control and Prevention (CDC) [14] have produced guiding principles for community engagement, which are similar and overlapping. While no standardised model for community engagement exists, the NIHR (principle donor of REDRESS) supports the UNICEF standards as a guide [26, 28]. The UNICEF [13] community engagement core standards are fundamental and cross-cut.

A literature review of 55 English-language studies examined the practical application of UNICEF’s core standards in community engagement within neglected tropical disease (NTD) research in low- and middle-income countries (Additional files 1, 2), with examples shown in Table 2.

**Table 2:** UNICEF [13] core standards and their application in NTD research

Core standard	Example in NTD research
Community participation	Qualitative studies provided deeper insights into community needs, enabling recommendations based on empirical findings. Informed design can be achieved through needs assessments and survey observations [29–31]. Stakeholder involvement in co-developing and implementing activities helps address concerns [32–35]
Empowerment and ownership	Studies highlight the inclusion of people affected by NTD [36, 37] and leader (religious or political) input in participant selection [36, 38, 39]. National-level engagement utilise existing structures and collaboration with MOH and local governance [29, 32, 40–42]. Projects operate at both national and community levels through advisory groups (ECLIPSE) [34], consortium workshops (ENDPOINT) [37], and participatory action research (COUNTDOWN) [33]. Community-level approaches include dialogue [43], and co-design workshops with social science expertise (ZEST) [31]
Inclusion	Inclusion was found in the involvement of snake bite victims as local champions [44] and data collection in areas of with high illiteracy and gender inequity [39] Purposive sampling was used to include vulnerable groups in data collections and within the research process such as persons affected [7, 30, 36, 37, 45–47], children and youths [31, 32, 48] or marginalised people [34]
Two way communication	Studies included time for feedback allocated in workshops [31, 37], feedback meetings for information sharing with questions and answers [41] and regular feedback from ministries and consortium partners (ASCEND) [29]. Mobile phones was used as communication pathway [42, 43]
Adaptability and localisation	Studies adapted data collection tools from findings of previous studies [7, 36, 49], situation analysis [37, 43] or pilot testing [30, 31]. The ECLIPSE decolonial approach is tailored to local countries, cultures, resources, and capacities [34]. Contextual adaptations [34], including linguistic appropriateness (e.g. Swahili, Igbo, Tshiluba, Macau, Portuguese) [30, 41, 43, 50], enhanced engagement
Building local capacity	Training communities as part of research process including training of trainers [37, 51], health workers [42, 52] or teachers [31] and community education [32] Peer researcher training on data collection with diverse actors, such as NTD staff, healthcare workers [30, 50], local staff [53], field assistants [41, 51], teachers [52], and community volunteers [43]

Participation was the most frequently applied principle, enabling communities to assess needs and contribute to research. Adaptability and localisation followed as the most applied standard, ensuring flexibility and contextual responsiveness. Empowerment emerged as both a process and an outcome, fostering community decision-making and ownership through leader involvement and project co-creation. Building local capacity leverages existing skills to promote self-sufficiency, often through training community members or peer researchers. Inclusion and two-way communication were the least commonly applied. The inclusion focused on engaging marginalised groups, although barriers persisted, particularly in the context of stigmatised diseases. Two-way communication, a key enabler of engagement, relies on mechanisms for clear instructions, feedback, data access, and information dissemination.

It was found in the review that research projects engaging communities generally did not incorporate all the UNICEF core standards, for example the project built on local capacity but did not include under-represented groups. This demonstrates a gap between idealistic community engagement principles and the reality of their application in research processes. This emphasises the need to include vulnerable groups through community engagement activities (such as participatory methods) within research cycles for their needs and voices to be heard as part of the decision-making process to ensure meaningful participation. Additionally, it highlights the significance of engaging with established social structures, such as religious or political leaders to foster collaboration. As a result, special attention was given to the value created from these engagements to improve health systems.

The REDRESS community engagement strategy closely aligns with the UNICEF core standards [13]. Some examples of their practical application include the participatory activities involving national MOH, county MOH, faith and traditional healers, as well as the inclusion of persons affected. Adaptability and localisation were ensured through formative baseline research informing programme modification. Capacity was built in research and NTDs at the national, county and community levels, with feedback processes in place for two-way communication. Participatory methods and the stakeholder involvement in decision making further promoted empowerment and ownership.

This study explores the value of community engagement within REDRESS for individuals aimed to improve care for people affected by skin NTDs in Liberia. Additionally, this research identifies challenges and areas of opportunity for strengthening community engagement and research within REDRESS, focusing on the involvement of MOH Liberia, co-researchers and peer-researchers. This is the first known study to explore community engagement activities related to skin NTD research within Liberia.

### Theoretical framework underpinning the study: value creation

Value creation is a process that turns resources and interactions into meaningful outcomes for communities and society [54]. Wenger et al. [55] identify key aspects of social fabric of learning, distinguishing between communities and networks, the latter referring to relationships and interactions.

**Fig. 1:**
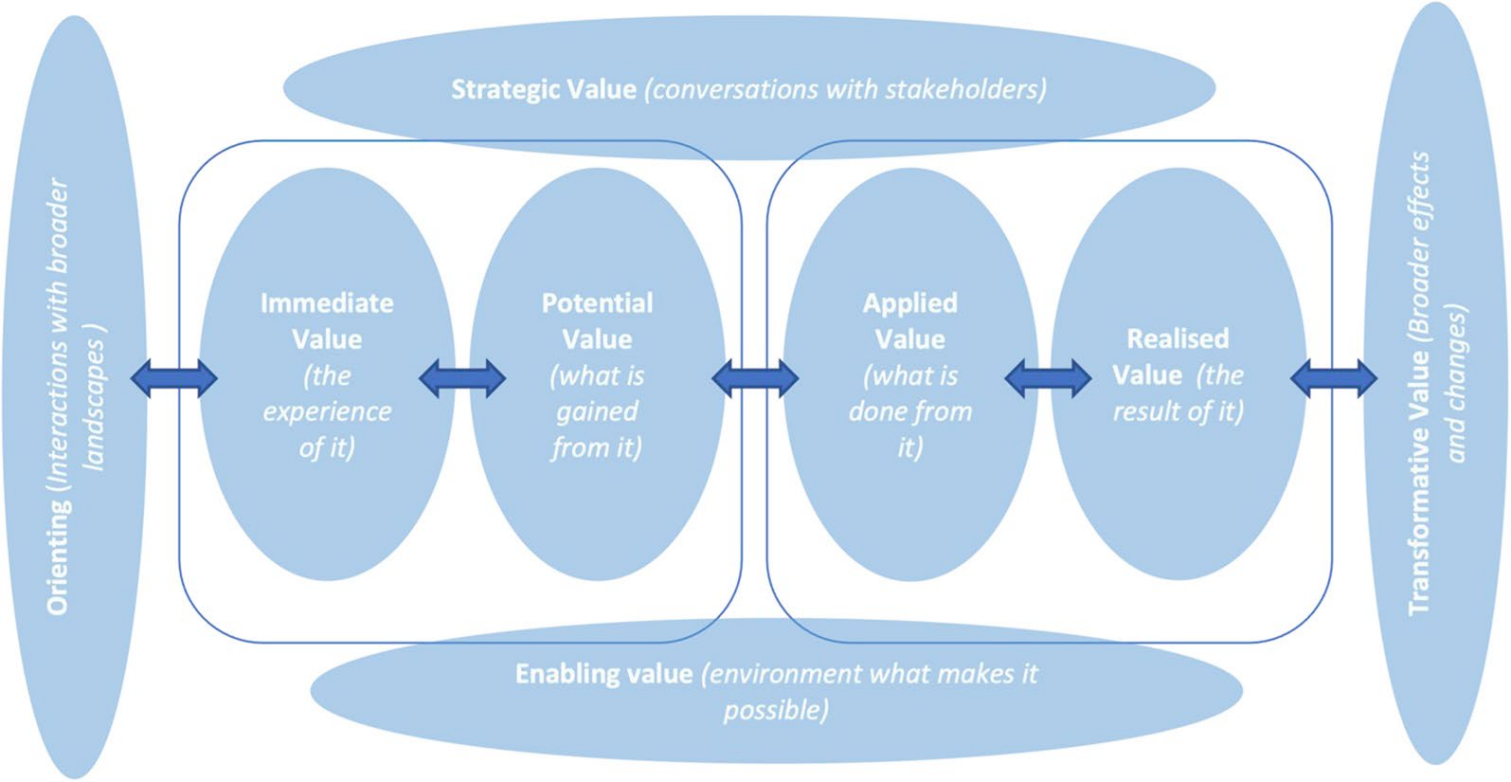
Schematic diagram of value creation cycles in communities, adapted from Wenger et al. (2011)

The value creation framework distinguishes interlinked cycles of value creation for communities and networks (Fig. 1) [55]. Framing narratives provide context for assessing value creation by clarifying the audience (value for whom) and perspective (short- or long-term impact). While personal narratives recount past events, they also reflect future aspirations and community-defined success. Although perceived future value is not a distinct cycle in the value creation framework, it offers insight into who it benefits and their perspectives. Immediate value emerges from the intrinsic worth of activities and interactions, whereas knowledge capital represents potential value that can be realised in the future. More broadly, knowledge capital encompasses human, social, tangible, reputational, and learning capital. Social capital value lies in relationships and reputations. When knowledge capital is adapted and applied in different contexts, it signifies a shift in practice and reflects applied value. However, changes in practice should not be assumed to lead to improvements; realised value assesses how applying knowledge capital impacts outcomes valued by stakeholders, including those adopting new practices. Reframing value is found when there is a reconsideration of what matters to stakeholders and how they define success, either for the individual or at the community level. Throughout this the strategic value refers to the engagements with relevant stakeholders.

The relationship between the cycles is complex and non-linear, there is no order or hierarchy to the cycles nor does one necessarily lead to another. Instead, they form a dynamic framework, offering a perspective on social learning that links activities to a spectrum of outcomes, which was drawn upon in this study.

## Methods

### Study aim and design

This study aims to explore the value of community engagement activities within a participatory action research study (REDRESS) to improve care for people affected by skin NTDs in Liberia. Specifically, it examines the engagement of co-researchers, peer-researchers, and Ministry of Health actors who play a dual role through their involvement in REDRESS.

A naturalistic paradigm shaped the study, exploring participants’ views and perspectives [56]. Qualitative methods were used because of the sensitive and deeply rooted nature of the topics discussed [57]. Primary data were gathered through semi-structured in-depth interviews (IDIs), which were triangulated with the analysis of 21 in-depth interviews with reflective diary participants.

### Data collection

#### In-depth interviews (data collected by MLP)

Eleven participants were interviewed in June 2022 across 3 locations: Monrovia (for national-level MOH), Lofa County and Margibi County (REDRESS intervention sites). Grand Gedeh County was the only intervention site excluded due to funding, time and challenging road conditions as the research was conducted during the rainy season.

The study focused on three communities engaged with REDRESS: peer-researchers (engaged in photovoice and reflective diaries), co-researchers and MOH Liberia dual roles (at the national and county levels). There was a range of participants involved with REDRESS throughout the study with varying extent of involvement, thus the interviewees most closely involved were purposively selected, with support for identification provided by the REDRESS research team, district health officers and NTD focal persons (Table 3). The representation of different types of participants meant that the small and information rich sample captured enough variations in perspective [58].

**Table 3:** Summary table of participants’ demographics for in-depth interviews

	Monrovia	Lofa	Margibi
Total	3	5	3
Gender			
Male	2	3	2
Female	1	2	1
Role			
Peer researcher	0	2	1
Co-researcher	0	3	1
Dual role (MOH)	3	3	2
Research experience			
Had been involved in research before REDRESS	3	0	0
First time involved in research	0	5	3

Topic guides were shaped by concepts of the Wenger et al. [55] value creation framework and then further adjusted as part of the iterative qualitative research process (Additional file 3). The interviews explored roles and responsibilities, hopes and expectations, past experiences, different influences, ethical considerations of a dual role, challenges, and recommendations. The interviews were audio recorded for 20 min to 1.5 h.

#### Reflective diary (data collected by the REDRESS research team)

Reflective diaries were kept by peer-researchers (formal health providers, informal health providers and persons affected (Table 4)) as part of monitoring and evaluation during the implementation phases to document short observations of the community and their work related to skin NTDs. The participants were chosen to reflect the diversity of persons involved in the study, because of their role. Twenty-five interviews and four focus groups were carried out with reflective diary participants during April 2022. Transcripts were purposively chosen on the basis of relevance, including content related to the value of participation, and captured a variation in demographics in roles and locations.

**Table 4:** Overview of reflective diary transcript participants

	Formal health provider	Person affected	Informal health provider
Lofa (IDI)	2 OIC, 1 CHSS, 2 CHV	2	2 TH, 2 FH
Margibi (FGD)	1 OIC, 2 CHA	1	1 TH, 1 FH
Grand Gedeh (IDI)	2 CHSS, 1 OIC, 1 CHV, 1 CHA	1	1 FH, 1 TH
Total number of participants	13	4	8

OIC, Officer in charge; CHSS/V/A, Community health service supervisor/volunteer/assistant; T/FH, Traditional/faith healer

### Data transcription and analysis

Audio recordings were transcribed and quality checked, with sections throughout all transcripts checked for accuracy and meaning. Taking an inductive epistemological position, information was built from the bottom up [59] via thematic framework analyses [60].

The lead researcher (MLP) familiarised with the data before presenting preliminary findings to REDRESS researchers in Liberia for their reflections, informing analysis and adding credibility. Iterative open coding developed a coding framework supported by NVIVO 12 which was then charted and themes emerged with narratives drafted.

Reflective diary transcript analysis followed a similar process using a coding framework developed in a participatory inductive process by Liberian researchers and co-researchers. These generated matrices were exported and interpreted following a grounded theory approach to generate social action theories [61].

Findings from the IDIs and reflective diaries were then compared to identify similarities and differences between methods and types of participants. The Wenger et al. [55] value creation framework introduced earlier was used to shape the overarching results analysis.

## Results

When exploring the value of community engagement activities within REDRESS, we identified five main categories structured around the value creation framework: perceived future value, immediate value, potential value, applied value, realised value and reframing value. Two overarching concepts are embedded within these themes: (1) participant position in research and; (2) creating an enabling environment.

### Perceived future value

The most described perceived future values related to involvement with REDRESS include capacity building, building networks and rapport with others, being able to help others and receiving resources.

All the participants wanted to achieve personal gain from participation, with capacity building being the most mentioned, including an impact on knowledge and research skills. The reflective diary participants expressed wanting NTD training: *“So that at least I will serve as a channel through which they can get treatment at the various facilities.”* (Faith healer, Lofa).

Learning how to engage and build rapport with different people was voiced by both persons affected and community health workers. A few participants (both the National MOH and persons affected) mentioned career development and improvement: “*Yes, so I feel that being part of the solution is going to help my career one [first] and I’ll be doing a great help to my community and my country*” (National dual role, Monrovia).

Personal gain was always described in addition to helping others: “*Perhaps my own will finish but other people, because there are plenty people that got this problem. We plenty*.” (Person affected 02). The IDI participants described this as being fulfilled by producing meaningful data and making recommendations to the MOH, and reflective diary participants through better treatment, awareness, referrals and knowledge dissemination.

Receiving resources was more frequently discussed by persons affected, such as a participant expecting to receive treatment and saying others had felt the same. Another person affected peer-researcher hoped that the study would make the county health team aware of their financial constraints. Finance was also mentioned by a co-researcher as appreciated, although it was not their primary expectation. There were also perceived future concerns, including time management and work efficacy for national level participants *(who undertook their REDRESS role alongside their MOH duties)*. A health worker expressed worries about using a digital tablet for the first time.

### Immediate value: activities and interactions

The immediate value included revelation and understanding about NTDs, improved collaboration with a range of actors, increased knowledge and improved social capital and connections. Linked to the immediate value are the enabling processes that facilitate activities, such as training and resources.

#### Activities leading to revelation

The co-researchers received training before and after the activities. All the participants reported learning about the burden of skin NTD, particularly their understanding of what persons affected go through as impactful for their roles as health workers. One co-researcher found “*the way in which the clients* (persons affected) *are being handled* (treated) *by the relatives or their community member”* (Dual role, Lofa) as the least enjoyable part of their involvement with the research*.* A person affected was shocked and frustrated that other persons affected with similar conditions were still hesitant to seek healthcare.

The majority of county-level IDI participants reported that travelling for data collection to different places and seeing other counties was a positive experience, allowing them to see rural areas and facilitating access to care for persons affected. Some female participants learned about their own cultures, particularly the roles of faith and traditional healers, with opportunities to actively engage with them. Participants from all levels felt that they had increased understanding of the health system and were surprised that the facility ledgers had gaps in record keeping *(records kept for NTD surveillance and reviewed in the REDRESS baseline survey).* Other health system surprises described by dual role participants were to do with actual administrative coverages and health worker salaries.

#### To collaborate with researchers and others

Collaboration with others was expressed by all IDI participants through learning and sharing of ideas. Persons affected felt proud to be regularly interacting with health workers, which was further elaborated by an interviewee who described having experienced isolation from society following their condition and how working with people and interacting with them made them feel *“very fine” (*person affected 03)*.*

Engagement at the institutional level was described by all participant groups. The national dual role participants explained how partnerships and department coordination facilitated through REDRESS had strengthened the interventions developed. County co-researchers sought support from colleagues to take on their work responsibility during research windows. Informal health providers from the reflective diaries also expressed their support and their desire to collaborate with the formal health system.

All the researchers described teamwork. Data collection was performed as a team that included co-researchers together with a more experienced research fellow. Roles included informed consent and a debriefing process afterwards, which demonstrated respect between different types of researchers. *“Yes, we used to discuss it … REDRESS people they actually know how to take care of somebody, they won’t act big like ‘I’m the boss’, no they will work together, and they will act like people doing the same thing.”* (Co-researcher, Lofa).

The community advisory board was described by co-researchers who were also board members. The board elected members to fulfil key positions within it (such as a person affected being elected as vice-president). *“I won the election overwhelmingly because I am a patient affected person and I am worth to work with them.”* (Person affected 03*).* The board worked together to discuss REDRESS and provide advice on actions.

Most participants talked highly about international collaboration with researchers and Master’s students from the United Kingdom. This included supporting research activities for students and mentoring their academic writing and research skills.

### Potential value: knowledge capital

Potential value was found when participants reported that they gained knowledge in research and NTDs and how to share that knowledge. All the IDIs with persons affected mentioned learning more about their own conditions and feeling that they could share this knowledge with others. Although one person affected still felt that their condition was a traditional sickness, they had learned that their condition could be cured. They expressed finding engagements with REDRESS very encouraging not to lose hope and that treatment would come.

Communication skills were seen as the primary skills learned by co-researchers, especially those holding a community health worker role in their routine work. They emphasised the importance of talking *“not harsh”* and asking patients about their conditions *“in a special way”* (Co-researcher, Lofa). Additionally, one co-researcher gained confidence in talking to more senior colleagues, and a person affected said that they learned to engage with the community.

The national dual role participants reported learning academic skills guided by mentors with feedback opportunities, which would be transferable and applicable to future jobs and projects. A few mentioned opportunities to contribute as co-author in published papers and a small number from Lofa County learned about new technology (phones and tablets).

#### Social capital

The interactions with faith and traditional healers were felt to be necessary because of the health-seeking pathway of the persons affected, who often sought care from these informal providers. Following the involvement of faith and traditional healers through participatory methods (photovoice and reflective diaries), a new friendliness with healers was described comparing it to a barrier being broken:

“*The faith healers were meaningful to me in the sense that, after they came up to tell us what they can do, and they promised us that if they happen to come across any case like that they will be able to communicate with the OIC* (officer in charge) *or the Nurse to the health facility to be able to receive them. It was really, really meaningful to me.”* (Co-researcher, Margibi). This engagement seemed to be supported by reflective diary discussions with faith and traditional healers who expressed positive feelings and expectations of future referrals.

“*And we also taught them* [healers] *the idea of relating with the health workers. And we also make the health workers to understand that these people, these traditional healers, they are part of us, they work in the health system”* (Co-researcher, Lofa).

### Applied and realised value: changes in practice

The participants described new friendships and improved relationships with persons affected by NTDs, as well as acting as potential change agents, by encouraging colleagues to improve the care they provide for persons affected.

#### Improving relationships with persons affected

The participants felt that they had made new friends from this process. Several IDI participants had improved relationships with persons affected and regularly checked up on them on the phone, one health worker added, *“they become family”* (Co-researcher, Lofa).

The co-researchers felt that the interactions with persons affected led them to feel happier and to hope. This was critical because to the stigmatisation and discrimination they face can lead them to *"feel unwanted in society”* (National dual role, Monrovia). Many participants voiced this and iterated the positive impact the activities were having on persons affected in the community and their health-seeking behaviour. This was mirrored by the persons affected, who proudly felt that they were leading others to be healed through their role. However, at this stage in the study (prior to roll out of the intervention) persons affected still experienced rejection within their community.

When asked about changes in trust, the majority of interviewees believed it had improved, with persons affected hiding less and traditional healers referring more. This was illustrated by the eagerness of persons affected to participate in research during the second data collection. The persons affected emphasised that the lack of free treatment (prior to intervention) hindered trust in the health system.

#### Changes in personal activities

Some participants expressed changes in the way they worked and counselled others. For example, three county dual role participants encouraged other health workers “*to get on their feet”* and refer cases (Co-researcher, Lofa). Three participants mentioned a change in their lifestyle activities: cleaning their home and encouraging others (person affected), using new technology capacity skills to download and save documents on mobile devices (health workers) and travelling to rural communities regularly to spread awareness (person affected).

### Reframing value: redefining success

The participants described a sense of having increased opportunity to influence change in care for persons affected, as well as the chance to grow professionally through academic opportunities.

The activities led to a change in attitude and the development of new academic interest from co-researchers and dual role participants. A few IDI participants aspired to develop publications and pursue health education: “*REDRESS have motivated me to take that path.”* (co-researcher, Lofa).

Research has been said to “*help broaden the mind*” (National dual role), and almost all county dual role participants expressed a change in their understanding of community needs (Table 5).

**Table 5:** Quotes illustrating a change in understanding of community needs

Quotes from participants holding a healthcare role at county level and taking part in research
“The most important, it made you to know things… It made you to know community, it made you to know people’s problems. Yes, problems that people going through, it will make you to know that also. It helped me”. (Co-researcher, Margibi)
“The research has given me more reason and power to do more on the NTDs… and it also showed me that these people we need to pay more attention to them, so it helped me…” (Co-researcher, Lofa)
“…REDRESS have made me to know that they have so many people out there that need help, they actually need help. And also made to know that, yes, those people that are there, we do not need to abandon them, to neglect them no, the need for them to come closer to us, and they need to get the rightful treatment so that they can get back on their feet like us”. (co-researcher, Lofa)
“Because it was like myself going into the community… it brought a kind of knowledge to me, that I’m not going into, right? That research alone made me to know that people are in the community they need help”. (Co-researcher, Margibi)

The participants generally thought their voice and ability to influence care for people affected by skin NTDs had changed, alongside a greater understanding and appreciation of the challenges experienced by persons affected by skin NTDs. A health worker was very insistent and quick to answer *“Yes, yes, yes. Yes. It got the power to change…the way in which we talk to them, encourage them, put yourself in that same position.”* (Co-researcher, Margibi), several health workers also agreed.

The persons affected saw change as a gradual process: *“it* (voice and ability to influence care for people) *has not changed, but it is changing*” (Person affected 01). The change in their voice and ability to influence was due to them feeling they were living proof that NTD treatment works and that drugs are effective.

### Participant position in research

The participant position in research forms part of what makes these processes possible (enabling value) as well as orienting value within the broader landscape. Dimensions of orienting value from the participants’ roles in research can be seen when their positions shaped their roles in REDRESS and their levels of influence.

#### How their position shaped their role in REDRESS

MOH participants explained that skills and knowledge helped them interact with patients in the field, and some county dual role participants stressed that their medical knowledge allowed them to easily identify cases: *“I play with medicine on daily basis”* (Co-researcher, Lofa), with similarities in activities between REDRESS and the MOH in collecting data and planning.

Some interviewees felt their input was crucial for enabling activity progress. For example, national MOH involvement in project implementation or co-researchers being able to speak the local dialect in data collection.

“*And in our absence (…) we will think how would that change be made? If there was nobody in between a project in the ministry?” “It’s* (recommendations from research findings) *happening simultaneously as we go along.”* (National MOH, Monrovia).

#### The influence participants felt on structures

There was a range of influence according to participants’ backgrounds and wider power structures, but across all participant types, participants expressed having the ability to influence others (including REDRESS decisions, MOH actors, and community members). The influence on REDRESS varied across counties and national MOH level participants felt they provide suggestions and play a part in the research process, with one participant mentioning the influence of the materials and questionnaires.

A participant (part of the CAB themselves) felt that the CAB was listened to by REDRESS in raising their concerns and providing guidance. *“We will be advising REDRESS; ‘o REDRESS this is what we want you people to do. This is the area we want you spend money. This is what we want you to pay attention to because this is the area that is falling. We want you to redress it’.”* (Person affected 03).

Surrounding the MOH, advocacy was felt more strongly by national-level participants, who were able to push for changes in their specific division. County MOHs roles reported they could advocate for community health during community meeting health talks. A person affected felt they held the ability to influence national MOH actors: “*Even if I go right now to the Ministry of Health, especially to the NTD department and tell them that I am a survivor, I am a patient affected person and I want to talk to the Minister, they will welcome me, definitely. Yes, so it is very good for me and I have a lot of influence.”* (Person affected 03).

Most of the interviewees felt that they could influence others, the term “*encourage*” was used several times by participants in counties, which was related to helping them participate in research and seeking healthcare. This was facilitated by NTD knowledge, good communication and effective drugs. A small number of county dual role participants felt able to give hope but unable to influence discussions.

#### Ethical considerations and their position

The concepts of “*consent*” and “*confidentiality*” were reflected on differently by the participants; for one participant, it was their first time learning more about this, whereas another participant felt that their position might “*push*” others, and thus did not collect data because their positionality could influence their responses. Other interviewees did not feel that their role had an influence on the consent process at the community level but had mixed views on their influence at the facility level.

*“…when you visit …. a community, as long as that thing has to do with health, majority of the people they are interested. So, despite your introduction, they are very keen… in their mind you brought solution and that solution, most of them, they think of medication, few of them think of food, and then few think of money.”* (National dual role, Monrovia).

Two stories were shared where dual role researchers had a potential conflict of interest on the basis of their observation of something that required intervention as part of their MOH role during data collection activities. The participant had a clear approach to manage both aspects ethically: “*So I’ll go ahead and do my findings, that is addressing the research questions, everything with that patient. After that, then I can encourage the patient along with the relative.*” (National dual role, Monrovia).

An interviewee reflected on their influence on data and explained the importance of being open-minded and objective and limiting biases: "*What I heard, what I saw, what I observed, was what I documented*” (National dual role, Monrovia).

### Creating an enabling environment

The enabling environment is due to processes with enabling value, which were described extensively by participants and have been described throughout the results. There were also a much smaller number of challenges described, which will also be considered to provide opportunities for growth and improvement in community engagement practices.

The main challenges described by participants related to the following:

1. Workload, particularly for national-level participants. These issues were mitigated through work plans and task sharing.
2. Challenges were encountered during data collection which made it less enjoyable. These included empty facility ledgers, difficulties in reaching facilities due to poor road conditions and long distances, language barriers, and participants being late for scheduled meetings.
3. Participant selection challenges were encountered during the first round of data collection when health representatives who support participant identification allegedly selected participants on the basis of on personal connections. This led to cancelled and rescheduled focus groups.
4. Insufficient travel reimbursement was described during the initial stages of data collection when the amount of gasoline support for follow-up activities was still being determined.
5. Diversion from the original objective. One participant emphasised the importance of reminding the team to maintain the original project objective and avoid too many adjustments.
6. Pre-existing challenges, including difficult patient access to treatment, patient transport issues (distance and costs), community health beliefs, gaps in knowledge about NTDs and supply chain gaps (which may lead patients to seek care from informal providers).

#### Proposed recommendation from participants

The IDI participants were asked for recommendations to ensure the best outcomes for researchers and the study. They emphasised the need for (1) more capacity building including mentorship, particularly in research and on NTDs; (2) knowledge dissemination and future research involvements; and (3) additional financial support.

For the REDRESS recommendations, participants wanted to improve patient care by (1) providing a consistent supply of medicine; (2) training more health workers; (3) raising constant and intensive awareness such as radio advertisements (which were included later within the intervention); (4) maintaining emphasis on sustainability and government ownership; and (5) including of community people and health workers as part of finding the solution.

*“I’ve seen programmes that came and people were doing very great. But as soon as that programme closes, or the project ends, everything goes back to zero.”* (National dual role, Monrovia).

## Discussion

Research has revealed that community engagement activities lead to value creation cycles by building capacity and improving relationships and providing communities tools to influence change. Value was found in community positionality by enabling processes that would not have otherwise taken place, but this came with ethical considerations. Sub-themes aligned with those of Belone et al. [62] cross-cutting community partnership constructs of trust, capacity, co-learning and power. REDRESS community engagement activities were designed to align with all 6 UNICEF [13] core community engagement standards and strongly promote the inclusion of persons affected. Additionally, this study highlights the importance of traditional and faith healer interactions, who hold trusted positions within the community and play a vital role in caring for people with skin NTDs.

### In terms of value creation

Overall, this study’s results resonate with Wenger et al.’s [55] conceptual framework promoting value creation for social learning. Aspiration narratives are built from perceived future value which defines what communities consider success.

*The immediate value* from engagement is the most basic cycle and emerges from activities and interactions, such as exposure to new ideas and places. The CDC (2011) understood the point of view of “insiders” as a key aspect of effective community collaboration through recognition and exposure to one’s own culture and health beliefs [14]. This was seen at an individual level when participants described new revelations from working together with persons affected, working in different geographic areas and at a larger scale with engagement activities informing project design. This ensures the relevance and identification of gaps and issues that need to be addressed for the project. The involvement of local stakeholders in informing and guiding research has also been successful in other NTD projects, such as ECLIPSE and ENDPOINT [34, 35]. The ENDPOINT (“Excellence in Disability Prevention Integrated across NTDs”) implementation research study took place in selected districts of the Awi Zone, North-West Ethiopia and assessed the integration and expansion of a holistic package of care into regular health services for individuals with lymphoedema due to podoconiosis, lymphatic filariasis, and leprosy [35]. The ECLIPSE research program uses a decolonial approach that involves different community engagement models in Brazil, Ethiopia and Sri Lanka to improve the physical and mental health outcomes of individuals with cutaneous leishmaniasis [34].

*The potential value* is demonstrated through the skills learned reflecting the various roles and processes in place for support and personal development. This allowed for two-way communication and information sharing, two of the UNICEF [13] core standards. Furthermore, sustained capacity building is seen in the training of co-researchers and peer researchers, as well as knowledge dissemination, fostering ownership and empowerment. Additionally, providing communities with new skills and resources led to a transformed ability to learn.

The training of trainers in global health partnerships has great potential for sustainability because it involves developing skills quickly and cost effectively; however, the literature shows that this training is influenced by factors such as time and resources [63]. Although these factors were not considered limitations from participants in this study, improving the cascade training of research activities provides high-quality training, allowing those receiving the final instructions to be clear in terms of roles and expectations and ensuring maximal benefit and value.

Social capital development was seen through collaboration and relationship building with persons affected, health providers and other researchers during research activities such as data collection. Tembo et al. (2021) reported that mutual interactions in co-production of health research promote relationships and trust [64]. This is essential in the context of Liberia given the complex health-seeking pathways of persons affected by skin NTDs, involving care-seeking from informal providers, as well as traditional beliefs and the stigma of skin NTDs [65]. Newly published research on collaboration with faith and traditional healers within REDRESS revealed that it contributed to earlier case detection and enhanced treatment outcomes [66]. The interactions between REDRESS, the MOH and traditional systems resulted in an observed change in the ability to influence care, which was found to be empowering.

Time management was the main anticipated and current challenge for the national MOH, who balanced both role responsibilities. This was also found to be a challenge for dual role researchers in Cole et al. [67] participatory research study; however, this challenge was offset by the benefits of participation, including capacity building and new partnerships. It is essential to listen to researchers’ concerns and for senior management teams (projects and government) to seek to understand these concerns and address them together.

A few participants voiced expectations of medications or more financial aid from their engagements. Flicker et al. [68] discuss ethical issues surrounding peer-researchers and find that projects should be cautious not to create false expectations beyond their capacity, as these expectations can strain partnerships and affect future engagements. This furthers the need for honest and transparent communication**,** with project objectives and participation implications made apparent to all to ensure realistic expectations. This applies to research participants as well as those holding a dual role position. Some recommendations made by participants reflected Liberia’s limited human resources and medication supply chain healthcare challenges [20, 26, 69, 70]. Capacity and financial constraints were shown to limit community engagement efforts related to a rabies programme in Tanzania, reinforcing the need for informed design of local resources [71].

Shifting to a long-term perspective, the *applied value and realised value* are related to changes in practice and performance improvement, respectively [55]. Through REDRESS community engagement, participants felt that their inputs and contributions—either through the CAB, as co-researchers, or as MOH staff—directly influenced and adapted the study on the basis of research findings. This exemplifies opportunities for change and flexibility, a key characteristic of action research such as REDRESS. This embedded change is in line with Ozano et al. [33] guiding principles for participatory health research which include equitable engagements, flexible action planning, addressing power differentials and ongoing learning. This participatory action has been reflected upon by REDRESS [72], who identified a 10-step journey for empowerment at the community level, covering health, society, mental wellbeing and livelihood, mapped by the WHO “*Strategic Framework for integrated control and management of skin-related Neglected Tropical Disease (NTDs)*” [3].

Behavioural changes from persons affected and informal health providers show how knowledge leads to reflections and actions, powered by self-determination. Martin et al. [43] similarly found that community engagement through a community dialogue approach helped communities to understand and address NTDs, improved medication compliance and changed health behaviours related to schistosomiasis in Mozambique.

The results revealed that community engagement activities promoted trust and health seeking behaviour among persons affected which was enabled by communication, confidentiality, and positionality. REDRESS reflected on this as part of the final evaluation, with the importance of lived experience between co-researchers and other persons affected emphasised [73]. Han et al. [74] explored community engagement experiences, and found that building trust is a crucial aspect of research ethics and effectiveness, but revealed problems in misaligned research priorities and poor communication of study results. Although not focused on skin NTDs, it supports recommendations for honest and transparent communication in projects seeking to engage communities. Notably, Wenger et al. [55] find importance in not assuming that realised value improvement is solely attributed to the community engagement but rather from a combination of factors.

*Reframing value* is a reconsideration of what is important to stakeholders [55]. The participants expressed learning about the disease burden and suffering faced by people affected by skin NTDs, which created a greater understanding of community needs and the need for help and encouragement of persons affected. This was evaluated at endline with health workers and CAB members both of whom described how their attitudes towards persons affected had drastically changed as a result of their involvement within REDRESS [75]. In addition to other activities, e.g. stigma training for health workers and informal providers and the establishment of peer support groups, these community engagement activities have contributed to persons affected re-joining society and contributing to the life of their communities [75]. The literature focusing on non-communicable diseases in LMICs reinforces that increasing local research capacity strengthening is crucial to combat disease, as they are the most suited in understanding their culture and context [76].

*Strategic value* was found in community positionality, which was a strength of this study. Traditional and faith healers who kept reflective diaries have contact with persons affected by skin NTDs and thus have valuable insights, and their longstanding and respected role plays an important role in health-seeking [44, 65]. REDRESS developed an intervention to sensitise faith and traditional healers about skin NTDs, including dispelling myths, identifying and referring NTD cases, providing basic psychosocial support and reducing stigma. Respect and recognition of roles were prioritised and key to building relationships and collaboration between formal and informal providers [66].

REDRESS includes vulnerable groups as peer researchers, with one person affected acting as co-researcher (collected data and ran research activities), which no studies from the literature review mentioned previously. This study’s results demonstrated inclusion of vulnerable groups, which is part of the UNICEF [13] core standard. This could have been further strengthened with the inclusion of more persons affected as co-researchers. However, the depth and quality of engagement with the sole person affected as co-researcher places it high on the participation ladder [77]. Over time, this participation has expanded to include the engagement and advocacy of persons affected within national and global forums [72].

Barriers to inclusion can be found when involving stigmatised diseases or sociodemographic groups. Moos et al. [44] scoping review highlights the importance of understanding the disease and social context essential for engagements, as well as ensuring that it is someone who is trusted and respected by the community carrying it out in an enabling area such as private locations.

Furthermore, a participatory study on lymphatic filariasis in coastal Kenya revealed that institutional barriers that make environments inappropriate for some groups, such as holding participatory meetings in the commissioner’s office, should be addressed [41]. However, in this study, the persons affected were passionate about engaging in activities, viewing themselves as proof of possible change, without raising institutional limitations to their engagement.

The results revealed participants felt that the CAB provided meaningful guidance and accountability to REDRESS. The CAB can be a power sharing mechanism ensuring localised and informed project design [64]. The ECLIPSE project established community- and policy-level advisory boards, including people affected by cutaneous leishmaniasis, and viewed the CAB as inwards-facing process focused on activity implementation [34]. A qualitative study exploring the meaningful engagement of advisory boards for women with breast cancer revealed gaps in patient knowledge and facilitation style as participation barrier in advisory boards [78]. Conducting reflection during the collaboration was suggested to alleviate barriers, promote relationship building, facilitate co-learning and improve researchers’ ability to meaningfully involve stakeholders [78].

Value creation cycles can be seen where problem recognition and the ability to change lead to a transformative effect. The value creation conceptual framework recommends the use of quantitative indicators to complement stories to assess the meaningful connection between them, which was not done in this study [55]. Community engagement was included as part of the final study evaluation and will be reported elsewhere; preliminary findings have been described in the REDRESS case study [72]. Preliminary findings from our study carried out around the time of baseline, before roll out of the REDRESS intervention, suggest that improved health outcomes can appear with increased community engagement, where activities lead to shared leadership and robust partnership structures [14]. This is essential for the sustainability of interventions introduced beyond REDRESS.

### Ethical considerations

REDRESS involves communities throughout the project cycle, so ethical issues have been extensively considered by the study team on a regular basis [79]. IDI national-level participants with high REDRESS involvement expressed more MOH advocacy suggesting decision-making power through shared leadership on the engagement continuum [14]. Some interviewees were unsure of the impact of their involvement in REDRESS which could explain why they felt they had less influence, although interpretations of the term *“influence”* varied.

Playing dual roles in research has been shown to create conflicts in other literature, such as roles not being fully disclosed or perceived obligations to participate [80, 81]. In this study regular safeguarding discussions included discussions about power dynamics, and the need to obtain informed consent was prioritised. The literature highlights that confusion surrounding confidentiality can be heightened if the participants know the researcher’s clinical background [80]. However, confidentiality was not raised as a concern, and the importance of trust with participants was emphasised.

A potential conflict of interest was illustrated when researchers acted as MOHs actors while researching, raising questions on how to navigate this scenario. Dean [7] described similar ethical dilemmas in which a participant with Buruli ulcer was not able to access treatment; thus following the interview, treatment was sought while working alongside the national NTD team. Brody and Miller [82] reported that tensions can be alleviated by the “*difference position*”, where clinicians’ and researchers’ activities are distinct enough to follow different ethical principles. Roles should be clearly defined to the participants, and reflexivity has been recommended in other studies to understand how dual roles influence consent [80, 81, 83]. The awareness of power dynamics can be achieved through practising reflexivity; thus, projects should seek to create spaces for this to take place. The relationship between NTDs and mental distress in Liberia has been studied elsewhere, which explores the impacts of power, privilege and identity experienced by person affected [84]. Further data collection regarding community engagement has taken place since this study, with the hope of building upon this work in the future and exploring further understanding of community engagement including power dynamics.

## Study limitations

The positionality of the lead researcher (MLP) as a young white female may affect interviewees’ perception; however their position as an outsider allows for fewer personal biases. Additionally, the lead researcher discussed the findings and analysis with Liberian researchers to promote correct interpretation of the findings.

There may be a gender bias as the sample was male dominated but this was seen as unlikely as both genders had similar views. MOH roles are more often filled by men, and more than half of co-researchers within REDRESS are men.

One intervention site was excluded from the study (Grand Gedeh county) because of funding, time and challenging road conditions as the research was conducted during the rainy season, however, the results would likely align with other locations’ views because similar community engagement activities were conducted.

## Conclusion

Skin NTDs affect a person’s physical and mental well-being, and the surrounding stigma and discrimination make community engagement critical to studies involving persons affected by skin NTDs. Furthermore, participatory action research and capacity building are central to REDRESS [18]; thus, recognising community engagement as meaningful and valuable is crucial. Community engagement activities create value, leading to meaningful empowerment, ownership, sustainability, and partnership, leading to broader health outcomes. The study identifies five areas of opportunity that would further facilitate community engagement activities within REDRESS and that are applicable to other research processes seeking to engage communities, particularly those with a dual role. These include the following: (1) improving the cascade training of research activities; (2) clearly defining roles; (3) engaging in honest and transparent communication; (4) acknowledge and address power; and (5) listening to researchers’ concerns.

The findings also reflected broader health system challenges, such as limited human resources and medication supply chains. Health system strengthening is part of the REDRESS [26] objectives, reinforcing the need for an enabling environment, as health system weakness can undermine the value of community engagement activities. The research findings were considered and incorporated within REDRESS, with further research conducted with CAB members, co-researchers and faith and traditional healers reflecting on their involvement and collaboration as part of REDRESS.

While findings are context specific, lessons learned on value creation apply to wider research seeking to engage communities, particularly those involving persons affected by stigmatised conditions and diverse community health beliefs. To our knowledge, this is the first study exploring the value of community engagement related to skin NTDs in Liberia.

## Supplementary Information

Additional file 1: Table of studies included in the literature review focusing on community engagement related to neglected tropical disease in low-middle income countries. Details of country of focus study type, condition of focus and community involved.

Additional file 2: Table of the application of the UNICEF (2020) community engagement standards across studies related to neglected tropical disease in low-middle income countries.

Additional file 3: In-Depth Interview Peer-Research/Dual Role Topic Guide used for data collection.

Additional file 4: GRIPP2 Long Form.

## Data Availability

Data is provided within the manuscript or supplementary information files.

## Abbreviations

CAB: Community advisory board
CDC: Centers for Disease Control and Prevention
IDI: In-depth interviews
MOH: Ministry of Health
NGO: Non-governmental organisations
NIHR: National Institute for Health and Care Research
NTD: Neglected tropical disease
WHO: World Health Organisation
SSSDs: Severe stigmatising skin diseases

## Footnotes

1 List of collaborating partners: Liverpool School of Tropical Medicine, University of Liberia Pacific Institute for Research & Evaluation, Ministry of Health Liberia, Actions Transforming Lives, American Leprosy Mission—AIM Initiative, Queen Margaret University, Effect Hope and funded by National Institute for Health Research and ANESVAD.

## References

[1] Mitjà O, Marks M, Bertran L, et al. Integrated control and management of neglected tropical skin diseases. PLoS Negl Trop Dis. 2017;11(1):e0005136. 10.1371/journal.pntd.0005136. 28103250 10.1371/journal.pntd.0005136 PMC5245794

[2] Yotsu RR. Integrated management of skin NTDs—lessons learned from existing practice and field research. Trop Med Infect Dis. 2018;3(4):120. 30441754 10.3390/tropicalmed3040120 PMC6306929

[3] World Health Organisation (WHO). A strategic framework for integrated control of skin-neglected tropical diseases; 2021.

[4] Houweling TAJ, Karim-Kos HE, Kulik MC, et al. Socioeconomic inequalities in neglected tropical diseases: a systematic review. PLoS Negl Trop Dis. 2016;10(5):e0004546. 10.1371/journal.pntd.0004546. 27171166 10.1371/journal.pntd.0004546 PMC4865383

[5] World Health Organisation (WHO). Ending the neglect to attain the Sustainable Development Goals: a road map for neglected tropical diseases 2021–2030. Geneva: World Health Organization; 2020.

[6] Chandler DJ, Fuller LC. The skin—a common pathway for integrating diagnosis and management of NTDs. Trop Med Infect Dis. 2018;3(3):101. 10.3390/tropicalmed3030101. 30274497 10.3390/tropicalmed3030101 PMC6161075

[7] Dean L, Tolhurst R, Nallo G, et al. Neglected tropical disease as a ‘biographical disruption’: listening to the narratives of affected persons to develop integrated people centred care in Liberia. PLoS Negl Trop Dis. 2019;13(9):e0007710. 10.1371/journal.pntd.0007710. 31490931 10.1371/journal.pntd.0007710 PMC6750611

[8] Ali O, Mengiste A, Semrau M, et al. The impact of podoconiosis, lymphatic filariasis, and leprosy on disability and mental well-being: a systematic review. PLoS Negl Trop Dis. 2021;15(7):1–17. 10.1371/journal.pntd.0009492. 10.1371/journal.pntd.0009492 PMC8266075 34237079

[9] Germain N, Augustin M, François C, et al. Stigma in visible skin diseases—a literature review and development of a conceptual model. J Eur Acad Dermatol Venereol. 2021;35(7):1493–504. 10.1111/jdv.17110. 33428316 10.1111/jdv.17110

[10] World Health Organization. Ending the neglect to attain the Sustainable Development Goals: a strategic framework for integrated control and management of skin-related neglected tropical diseases. Geneva: Switzerland; 2022.

[11] Tindana PO, Singh JA, Tracy CS, et al. Grand challenges in global health: community engagement in research in developing countries. PLoS Med. 2007;4(9):e273. 10.1371/journal.pmed.0040273. 17850178 10.1371/journal.pmed.0040273 PMC1989740

[12] Glandon D, Paina L, Alonge O, et al. 10 Best resources for community engagement in implementation research. Health Policy Plan. 2017;32(10):1457–65. 10.1093/heapol/czx123[publishedOnlineFirst:2017/11/02]. 29092039 10.1093/heapol/czx123 PMC5886100

[13] UNICEF. Minimum Quality Standards and Indicators for Community Engagement, 2020.

[14] Centers for Disease Control and Prevention (CDC). Principles of community engagement. 2nd ed. National Institutes of Health (NIH); 2011.

[15] REDRESS partnership. Case study: participatory approaches, training and new relationships reduce stigma and violence experienced by persons affected by NTDs and promote participation and inclusion, 2024.

[16] Topp SM, Schaaf M, Sriram V, et al. Power analysis in health policy and systems research: a guide to research conceptualisation. BMJ Glob Health. 2021. 10.1136/bmjgh-2021-007268. 34740915 10.1136/bmjgh-2021-007268 PMC8573637

[17] Kagwanja N, Molyneux S, Whyle E, et al. How does power shape district health management team responsiveness to public feedback in low- and middle-income countries: an interpretive synthesis. Health Policy Plan. 2023;38(4):528–51. 10.1093/heapol/czac105[publishedOnlineFirst:2022/12/07]. 36472343 10.1093/heapol/czac105 PMC10089071

[18] REDRESS. REDRESS Community Engagement and Involvement Strategy, 2020.

[19] World Bank Group (WBG). Liberia Country The World Bank Data2022 [Available from: https://data.worldbank.org/country/liberia. Accessed 31 Aug 2022.

[20] Simen-Kapeu A, Lewycka S, Ibe O, et al. Strengthening the community health program in Liberia: lessons learned from a health system approach to inform program design and better prepare for future shocks. J Glob Health. 2021;11:07002. 10.7189/jogh.11.07002[publishedOnlineFirst:2021/03/26]. 33763217 10.7189/jogh.11.07002 PMC7956118

[21] Ministry of Health, Goverment of Liberia. Strategic plan for integrated case management of Neglected Tropical Diseases (NTDs) 2016–2020, 2016.

[22] REDRESS. Our Vision 2021 [Available from: https://www.redressliberia.org/about-us/. Accessed 1 Sept 2022.

[23] REDRESS. Our approach: REDRESS, Liverpool School of Tropical Medicine.; 2021 [Available from: https://www.redressliberia.org/our-approach/. Accessed 9 Dec 2021.

[24] REDRESS. Full detailed research plan. Liverpool School of Tropical Medicine 2020.

[25] REDRESS. Strengthening people-centred health systems for people affected by severe stigmatising skin diseases in Liberia: REDRESS, Liverpool School of Tropical Medicine; 2021 [Available from: https://www.redressliberia.org/. Accessed 9 Dec 2021.

[26] REDRESS. Executive summary: findings from county, facility and community key informant interviews, 2021.

[27] World Health Organisation (WHO). Community engagement: a health promotion guide for universal health coverage in the hands of the people. Geneva: World Health Organization; 2020.

[28] National Institute for Health and Care Research (NIHR). Community engagement and involvement 2024. Available from: https://www.nihr.ac.uk/researchers/i-need-help-funding-my-research/tips-for-making-your-application/community-engagement-and-involvement. Accessed 13 May 2024.

[29] Clark A, Hill B, Bannerman R, et al. Adapting an integrated neglected tropical disease programme in response to COVID-19. Trans R Soc Trop Med Hyg. 2021;115(5):441–6. 10.1093/trstmh/traa180. 33570149 10.1093/trstmh/traa180 PMC7928664

[30] Ukwaja KN, Alphonsus C, Eze CC, et al. Investigating barriers and challenges to the integrated management of neglected tropical skin diseases in an endemic setting in Nigeria. PLoS Negl Trop Dis. 2020;14(4):e0008248. 10.1371/journal.pntd.0008248[publishedOnlineFirst:2020/05/01]. 32352967 10.1371/journal.pntd.0008248 PMC7217480

[31] Person B, Knopp S, Ali SM, et al. Community co-designed schistosomiasis control interventions for school-aged children in Zanzibar. J Biosoc Sci. 2016;48(S1):S56–73. 10.1017/S0021932016000067. 27428066 10.1017/S0021932016000067

[32] Madon S, Malecela MN, Mashoto K, et al. The role of community participation for sustainable integrated neglected tropical diseases and water, sanitation and hygiene intervention programs: a pilot project in Tanzania. Soc Sci Med. 2018;202:28–37. 10.1016/j.socscimed.2018.02.016[publishedOnlineFirst:2018/03/05]. 29501716 10.1016/j.socscimed.2018.02.016 PMC5906643

[33] Ozano K, Dean L, Adekeye O, et al. Guiding principles for quality, ethical standards and ongoing learning in implementation research: multicountry learnings from participatory action research to strengthen health systems. Health Policy Plan. 2020;35(Supplement_2):ii137–49. 10.1093/heapol/czaa123. 33156936 10.1093/heapol/czaa123 PMC7646736

[34] Polidano K, Parton L, Agampodi SB, et al. Community engagement in cutaneous leishmaniasis research in Brazil, Ethiopia, and Sri Lanka: a decolonial approach for global health. Front Public Health. 2022. 10.3389/fpubh.2022.823844. 35242734 10.3389/fpubh.2022.823844 PMC8885625

[35] Davies B, Kinfe M, Ali O, et al. Stakeholder perspectives on an integrated package of care for lower limb disorders caused by podoconiosis, lymphatic filariasis or leprosy: a qualitative study. PLoS Negl Trop Dis. 2022;16(1):e0010132. 10.1371/journal.pntd.0010132. 35061673 10.1371/journal.pntd.0010132 PMC8809619

[36] Eneanya OA, Garske T, Donnelly CA. The social, physical and economic impact of lymphedema and hydrocele: a matched cross-sectional study in rural Nigeria. BMC Infect Dis. 2019;19(1):332. 10.1186/s12879-019-3959-6. 31014256 10.1186/s12879-019-3959-6 PMC6480436

[37] Tesfaye A, Semrau M, Ali O, et al. Development of an integrated, holistic care package for people with lymphoedema for use at the level of the Primary Health Care Unit in Ethiopia. PLoS Negl Trop Dis. 2021;15(4):1–13. 10.1371/journal.pntd.0009332. 10.1371/journal.pntd.0009332 PMC8086999 33878110

[38] Kisoka W, Mushi D, Meyrowitsch DW, et al. Dilemmas of community-directed mass drug administration for lymphatic filariasis control: a qualitative study from urban and rural Tanzania. J Biosoc Sci. 2017;49(4):447–62. 10.1017/s0021932016000365[publishedOnlineFirst:2016/07/30]. 27470198 10.1017/S0021932016000365

[39] Anyolitho MK, Poels K, Huyse T, et al. Knowledge, attitudes, and practices regarding schistosomiasis infection and prevention: a mixed-methods study among endemic communities of western Uganda. PLoS Negl Trop Dis. 2022;16(2):e0010190. 10.1371/journal.pntd.0010190. 35196328 10.1371/journal.pntd.0010190 PMC8865686

[40] Silumbwe A, Halwindi H, Zulu JM. How community engagement strategies shape participation in mass drug administration programmes for lymphatic filariasis: the case of Luangwa district, Zambia. PLoS Negl Trop Dis. 2019;13(11):e0007861. 10.1371/journal.pntd.0007861. 31774820 10.1371/journal.pntd.0007861 PMC6905562

[41] Njomo DW, Kibe LW, Kimani BW, et al. Addressing barriers of community participation and access to mass drug administration for lymphatic filariasis elimination in Coastal Kenya using a participatory approach. PLoS Negl Trop Dis. 2020;14(9):e0008499. 10.1371/journal.pntd.0008499. 32936792 10.1371/journal.pntd.0008499 PMC7494106

[42] Stanton M, Molineux A, Mackenzie C, et al. Mobile technology for empowering health workers in underserved communities: new approaches to facilitate the elimination of neglected tropical diseases. JMIR Public Health Surveill. 2016;2(1):e2. 10.2196/publichealth.5064. 27227155 10.2196/publichealth.5064 PMC4869228

[43] Martin S, Rassi C, Antonio V, et al. Evaluating the feasibility and acceptability of a community dialogue intervention in the prevention and control of schistosomiasis in Nampula province, Mozambique. PLoS ONE. 2021. 10.1371/journal.pone.0255647. 34351982 10.1371/journal.pone.0255647 PMC8341517

[44] Moos B, Williams D, Bolon I, et al. A scoping review of current practices on community engagement in rural East Africa: recommendations for snakebite envenoming. Toxicon: X. 2021. 10.1016/j.toxcx.2021.100073. 34381992 10.1016/j.toxcx.2021.100073 PMC8334718

[45] Guthmann JP, Calmet J, Rosales E, et al. Patients’ associations and the control of leishmaniasis in Peru. Bull World Health Organ. 1997;75(1):39–44. 9141749 PMC2486976

[46] Abdulmalik J, Nwefoh E, Obindo J, et al. Emotional difficulties and experiences of stigma among persons with lymphatic filariasis in Plateau State. Nigeria Health Hum Rights. 2018;20(1):27–40. 30008550 PMC6039724

[47] Collinson S, Frimpong VNB, Agbavor B, et al. Barriers to Buruli ulcer treatment completion in the Ashanti and Central Regions, Ghana. PLoS Negl Trop Dis. 2020;14(5):e0008369. 10.1371/journal.pntd.0008369. 32453800 10.1371/journal.pntd.0008369 PMC7274448

[48] Nwaorgu OC, Okeibunor J, Madu E, et al. A school-based schistosomiasis and intestinal helminthiasis control programme in Nigeria: acceptability to community members. Trop Med Int Health. 1998;3(10):842–9. 10.1046/j.1365-3156.1998.00313.x. 9809919 10.1046/j.1365-3156.1998.00313.x

[49] Legge H, Kepha S, Prochazka M, et al. Implementer and recipient perspectives of community-wide mass drug administration for soil-transmitted helminths in Kwale County. Kenya PLoS Negl Trop Dis. 2020. 10.1371/journal.pntd.0008258. 32310966 10.1371/journal.pntd.0008258 PMC7192516

[50] Mpanya A, Hendrickx D, Vuna M, et al. Should I get screened for sleeping sickness? A qualitative study in Kasai Province, Democratic Republic of Congo. PLoS Negl Trop Dis. 2012;6(1):e1467. 10.1371/journal.pntd.0001467. 22272367 10.1371/journal.pntd.0001467 PMC3260312

[51] Biritwum NK, Garshong B, Alomatu B, et al. Improving drug delivery strategies for lymphatic filariasis elimination in urban areas in Ghana. PLoS Negl Trop Dis. 2017;11(5):e0005619. 10.1371/journal.pntd.0005619[publishedOnlineFirst:2017/05/12]. 28493966 10.1371/journal.pntd.0005619 PMC5441634

[52] Gyapong JO, Webber RH, Bennett S. The potential role of peripheral health workers and community key informants in the rapid assessment of community burden of disease: the example of lymphatic filariasis. Trop Med Int Health TM & IH. 1998;3(7):522–8. 10.1046/j.1365-3156.1998.00266.x. 9705185 10.1046/j.1365-3156.1998.00266.x

[53] Marks M, Kwakye-Maclean C, Doherty R, et al. Knowledge, attitudes and practices towards yaws and yaws-like skin disease in Ghana. PLoS Negl Trop Dis. 2017;11(7):1–12. 10.1371/journal.pntd.0005820. 10.1371/journal.pntd.0005820 PMC5552343 28759580

[54] International Federation of Accountants (IFAC). Understanding value creation. New York: International Federation of Accountants; 2020.

[55] Wenger E, Trayner B, Laat M. Promoting and assessing value creation in communities and networks: a conceptual framework. 2011.

[56] Pope C, Mays N. Qualitative research: reaching the parts other methods cannot reach: an introduction to qualitative methods in health and health services research. BMJ. 1995;311(6996):42. 7613329 10.1136/bmj.311.6996.42 PMC2550091

[57] Ritchie J, Lewis J. Chapter 2: The applications of qualitative methods to social research in “Qualitative research practice: a guide for social science students and researchers.” London: Sage Publications Ltd.; 2003. p. 24–46.

[58] Kielmann K, Cataldo F, Seeley J. Introduction to qualitative research methodology: a training manual. Department for International Development (DfID); 2012.

[59] Blaikie N. Chapter 1: Major choices in social inquiry in “approaches to social inquiry.” Cambridge: Polity; 2007. p. 5–30.

[60] Gale NK, Heath G, Cameron E, et al. Using the framework method for the analysis of qualitative data in multi-disciplinary health research. BMC Med Res Methodol. 2013;13(1):117. 10.1186/1471-2288-13-117. 24047204 10.1186/1471-2288-13-117 PMC3848812

[61] Snape D, Spencer L. Chapter 1: The foundations of qualitative research in “Qualitative research practice: a guide for social science students and researchers.” London: Sage Publications Ltd.; 2003.

[62] Belone L, Lucero JE, Duran B, et al. Community-based participatory research conceptual model: community partner consultation and face validity. Qual Health Res. 2016;26(1):117–35. 10.1177/1049732314557084. 25361792 10.1177/1049732314557084 PMC4839192

[63] Mormina M, Pinder S. A conceptual framework for training of trainers (ToT) interventions in global health. Glob Health. 2018. 10.1186/s12992-018-0420-3. 10.1186/s12992-018-0420-3 PMC6198384 30348183

[64] Tembo D, Hickey G, Montenegro C, et al. Effective engagement and involvement with community stakeholders in the co-production of global health research. BMJ. 2021;372:n178. 10.1136/bmj.n178. 33593805 10.1136/bmj.n178 PMC7879275

[65] McCollum R, Berrian H, Theobald S, et al. Barriers and enablers to health-seeking for people affected by Severe Stigmatising Skin Diseases (SSSDs): a scoping review. Soc Sci. 2022;11(8):332. 10.3390/socsci11080332.

[66] REDRESS partnership. Beyond the biomedical: collaboration with faith & traditional healers in the management of skin NTDs is possible, leading to earlier case detection and stigma reduction, 2024.

[67] Cole CA, Edelman EJ, Boshnack N, et al. Time, dual roles, and departments of public health: lessons learned in CBPR by an AIDS service organization. Prog Community Health Partnersh. 2013;7(3):323–30. 10.1353/cpr.2013.0034. 24056514 10.1353/cpr.2013.0034 PMC4794330

[68] Flicker S, Roche B, Guta A. Peer research in action III: ethical issues. In: Community based research working paper series. Toronto, The Wellsesley Institute; 2010.

[69] Prochazka M, Timothy J, Pullan R, et al. “Buruli ulcer and leprosy, they are intertwined”: patient experiences of integrated case management of skin neglected tropical diseases in Liberia. PLoS Negl Trop Dis. 2020;14(2):e0008030. 10.1371/journal.pntd.0008030. 32023242 10.1371/journal.pntd.0008030 PMC7001903

[70] Agboraw E, Sosu F, Dean L, et al. Factors influencing mass drug administration adherence and community drug distributor opportunity costs in Liberia: a mixed-methods approach. Parasit Vectors. 2021;14(1):557. 10.1186/s13071-021-05058-w. 34711278 10.1186/s13071-021-05058-w PMC8555123

[71] Bardosh K, Sambo M, Sikana L, et al. Eliminating rabies in Tanzania? Local understandings and responses to mass dog vaccination in Kilombero and Ulanga Districts. PLoS Negl Trop Dis. 2014;8(6):e2935. 10.1371/journal.pntd.0002935. 24945697 10.1371/journal.pntd.0002935 PMC4063706

[72] REDRESS partnership. Empowering person affected by skin NTDs: REDRESS’ 10 Key Steps; 2024.

[73] REDRESS partnership. Involving a diverse range of community actors strengthens case detection for skin NTDs; 2024.

[74] Han H-R, Xu A, Mendez KJW, et al. Exploring community engaged research experiences and preferences: a multi-level qualitative investigation. Res Involv Engag. 2021. 10.1186/s40900-021-00261-6. 10.1186/s40900-021-00261-6 PMC8008581 33785074

[75] REDRESS partnership. Participatory approaches, training and new relationships reduce stigma and violence experienced by persons affected by NTDs and promote participation and inclusion; 2024.

[76] Malekzadeh A, Michels K, Wolfman C, et al. Strengthening research capacity in LMICs to address the global NCD burden. Glob Health Action. 2020;13(1):1846904. 10.1080/16549716.2020.1846904. 33373280 10.1080/16549716.2020.1846904 PMC7782223

[77] Arnstein SR. A ladder of citizen participation. J Am Inst Plann. 1969;35(4):216–24. 10.1080/01944366908977225.

[78] Han H-R, Xu A, Mendez KJW, et al. Exploring community engaged research experiences and preferences: a multi-level qualitative investigation. Res Involv Engag. 2021;7(1):19. 10.1186/s40900-021-00261-6. 10.1186/s40900-021-00261-6 PMC8008581 33785074

[79] Guta A, Voronka J. Ethical issues in community-based, participatory, and action-oriented forms of research; 2020.

[80] Hiller A, Vears D. Reflexivity and the clinician-researcher: managing participant misconceptions. Qual Res J. 2016;16:13–25. 10.1108/QRJ-11-2014-0065.

[81] Judkins-Cohn TM, Kielwasser-Withrow K, Owen M, et al. Ethical principles of informed consent: exploring nurses’ dual role of care provider and researcher. J Contin Educ Nurs. 2014;45(1):35–42. 10.3928/00220124-20131223-03. 24369754 10.3928/00220124-20131223-03

[82] Brody H, Miller FG. The clinician-investigator: unavoidable but manageable tension. Kennedy Inst Ethics J. 2003;13(4):329–46. 10.1353/ken.2004.0003. 15049297 10.1353/ken.2004.0003

[83] Geddis-Regan AR, Exley C, Taylor GD. Navigating the dual role of clinician-researcher in qualitative dental research. JDR Clin Transl Res. 2022;7(2):215–7. 10.1177/2380084421998613. 10.1177/2380084421998613 33618559

[84] McCollum R, Barrett C, Zawolo G, et al. ‘The Lost Peace’: Evidencing the Syndemic Relationship between Neglected Tropical Diseases and Mental Distress in Liberia. Trop Med Infect Dis. 2024;9(8):183. 39195621 10.3390/tropicalmed9080183 PMC11359536

