# Supplementary material for: Exploring the value of community engagement activities within a participatory action research study to improve care for people affected by skin neglected tropical diseases in Liberia
Source: Res Involv Engagem. 2025 Mar 24;11:27. doi: 10.1186/s40900-025-00695-2 (PMC11931812; doi:10.1186/s40900-025-00695-2)
Supplement: Supplementary file 3 — Additional file 3: In-Depth Interview Peer-Research/Dual Role Topic Guide used for data collection. [file 40900_2025_695_MOESM3_ESM.docx]

Title: In-Depth Interview Peer-Research/ Dual Role Topic Guide

Coded bleu: Only applicable to dual role interviews

MOH= Ministry of health
HCW= Health care worker
PA= Person affected

**Definition/ Role**

1. Please can you describe your role in MOH/HCW/community?
2. Please can you describe your role in REDRESS?
   - *What do you do? Who do you interact with? How much do you participate? How do you balance this workload?*
3. Overall feeling about this role?
   - *Which parts did you enjoy most? And least?*
4. What made you participate?
   - *Hopes and expectations? Concerns or worries? How has this been fulfilled so far?*

**Impact/value**

1. How does your participation affect your experience?
2. Please describe a meaningful activity that you participated in and your experience of it?
   - *New insight gained? Learnt anything new or surprising? How did this affect/influence you? Enable something? Contribute to REDRESS goal? Change your or someone else’s understanding of what matters?*
3. How did your MOH/HCW/PA role help your REDRESS role?
4. How has your role with REDRESS influenced your MOH/HCW role?
   - *Do things differently at work? Has your voice or ability to influence care for PA changed?*
5. Have you felt a change since you started?
   - *What was it? Why did this happen?*
6. How do you feel relationship with others have changed?
   - *Interaction with PA/ HCW/ REDRESS colleagues?*
7. Do you feel you can influence what happens?
   - *Why/ why not? On others? Within REDRESS?*
8. Have you felt like your role ever raised ethical considerations?
9. How do you feel your role MOH/HCW influences the consent process?
10. How might your MOH/HCW role influence discussion?
11. Is there anything you do differently compared to REDRESS non MOH/HCW staff?

**Challenges**

1. What kind of challenges did you have?
2. Did you have any experiences that you felt were difficult? How did you deal with this?

**Future**

1. Recommendation to ensure best outcomes for those working dual role?
2. Recommendation that would improve your own outcomes?
3. Recommendation to improve the outcomes of the study?
